# A trustful relationship—the importance for relatives to actively participate in the meeting with the physician

**DOI:** 10.3402/qhw.v8i0.20608

**Published:** 2013-05-20

**Authors:** Sandra Pennbrant

**Affiliations:** Division of Caring Sciences, University West, Trollhaettan, Sweden

**Keywords:** Elderly care, physician, qualitative interview study, relative, socio-cultural perspective

## Abstract

In previous research, no uniform picture emerged of the role of relatives in the meeting between an elderly patient and a physician. Knowledge about relatives’ experiences of the meeting between an elderly patient and a physician will help healthcare practitioners better understand the role of relatives during the meeting and how practitioners can assist relatives in assuming their supporting role more efficiently. The purpose of this study is to explore experiences of relatives of meeting with the physician in a hospital setting when an elderly patient is discharged from hospital care to home care, in order to identify aspects that may facilitate relatives in taking up their role in a more efficient manner. This descriptive and exploratory study is based on 20 interviews with relatives. The result shows that the physician's communication style influences the meeting between the relative, the elderly patient, and the physician, and that this style is the result of power and interaction. A trustful relationship during the meeting between the relative and the physician can increase the relative's feeling of confidence with the healthcare organization and treatment of the elderly patient. The relative has an important supporting role in the care for the elderly family member, both in the hospital and the home setting. It is likely that the relative's value as a resource, for both the patient and the physician, increases as the relative experiences feelings of confidence in the meeting with the physician. It is therefore of value to increase our knowledge about the conditions and circumstances facilitating and/or hampering the meeting between the relative and the physician. The result stresses the importance of encouraging relatives to participate in the meeting. Physicians need more guidance and training in communication skills, respectful demeanor, and collaboration while meeting the relatives.

Relatives are an important resource for patients (Paul, Hendry, & Cabrelli, 2004; Wilz & Meichsner, 2012), especially for elderly patients (Attree, 2001; Hertzberg, Ekman, & Axelsson, 2003). Relatives are often involved in the meeting between an elderly patient and a physician at the time of hospital discharge. The responsibility of relatives in Sweden has increased in recent years and is expected to increase further as patients are discharged earlier from hospital care (Swedish Government Bill, 2005/2006). This study focuses on relatives’ experience of the meeting with the physician in a hospital setting when an elderly patient is discharged from hospital care to home care in order to identify aspects that may help relatives take up their supporting role in a more efficient manner.

## Previous research on the meeting between relatives and physicians

Previous research has found that physicians are uncomfortable when a relative is present at the meeting as they feel relatives negatively influence the patient and may distract from the patient's needs (Mehnert, Lehmann, & Koch, 2012; Shields et al., 2005). Nevertheless, sometimes it is necessary to involve relatives in the meeting concerning an elderly patient's treatment and/or care (Korfage et al., 2013; Sayers & Bethell, 2004; Wolff, Clayman, Rabins, Cook, & Roter, 2012). In this regard, studies show that the majority of patients in mental health prefer that their relatives are involved in their treatment with the physician (Perreault, Paquin, Kennedy, Desmarais, & Tardif, 1999; Wolff & Roter, 2012), that the family is very important in cancer care (Beisecker, Brecheisen, Ashworth, & Hayes, 1996; Glasser, Prohaska, & Gravdal, 2001; Royak-Schaler et al., 2006; Schweitzer et al., 2005), and that patients and their relatives prefer a patient-centered approach during physician consultation (Attree, 2001; Dowsett et al., 2000).

Relatives have been found to value mutual confidence, dialogue and trust during the meeting with the physician (Attree, 2001; Schilling et al., 2002). Relatives find it problematic when they are unsure about their role and about whom to turn to in order to obtain information and support to decrease their level of frustration and risk of exhaustion (Kristjanson, 1999). One study revealed that relatives who were unable to express themselves experienced the care situation negatively as they felt they were invisible to the physician and received neither support nor information (Levine & Zuckerman, 1999). Another study found that communication is an important factor explaining levels of patient and caregiver coping and emotional distress (Kissane, 2004). Relatives tend to feel satisfied and secure when the healthcare staff supplies adequate information and when they are involved in the long-term planning for the patient (Magnusson & Granskär, 2005).

Previous research has often focused on relatives’ experiences of involvement in elderly patients’ healthcare in situations such as dementia (Fortinsky, 2001; Gilmour, 2002; Glasser & Miller, 1998; Hertzberg & Ekman, 2008), hospice care and long-term hospital care (Beisecker et al., 1996; Jakobsson, Bergh & Öhlén, 2007; Magnusson & Granskär, 2005; Royak-Schaler et al., 2006), and on caregivers accompanying patients to a care center (Silliman, 2000). In previous research, no uniform picture emerges of the role of relatives in the meeting between the elderly patient and physician. Some studies indicate that the elderly patient's role may be diminished by the relative's involvement (Greene, Majerovitz, Adelman, & Rizzo, 1994; Ho, 2008), while other studies indicate the opposite effect (Brown, Brett, Stewart, & Marshall, 1998; Glasser et al., 2001; Labrecque, Blanchard, Ruckdeschel, & Blanchard, 1991; Sakai & Carpenter, 2011; Shields et al., 2005; Wilz & Meichsner, 2012; Wolff et al., 2012). Possible explanations for this discrepancy could be that the role and effect of the relative depends on the situation, the relationship with the patient, the patient's state of health, or on the physician's demeanor toward both the elderly patient and the relative, or on the physician's willingness and ability to accommodate patients’ and families’ capabilities, needs, and preferences (Beisecker, 1989; Hesselink et al., 2012; Silliman, 2000). Knowledge about relatives’ experiences of the meeting between the elderly patient and the physician will help healthcare practitioners better understand the relatives’ role during the meeting and how practitioners can assist relatives in assuming their supporting role more efficiently. In this way, the relative can provide safety and support to the elderly patient.

### Theoretical perspective

The theoretical understanding is based on a sociocultural perspective (Säljö, 2000; Vygotsky, 1987) explaining the relationship between thinking and communication. Thinking can be understood as an internal discussion within the person by means of language. The link between the individual and the surrounding world presupposes communication between individuals and is based on thought (Säljö, 2000; Vygotsky, 1987).

Health and hospital care is characterized by situations where meetings between people are of crucial importance. The communication between relative and physician is an example of a meeting with various underlying unspoken views and expectations concerning the situation at hand. The collaboration between relative and physician does not take place in a social vacuum; on the contrary, it is part of a social whole. The health and hospital care takes place in a sociocultural context, which means that skills, insights and knowledge are shaped by the prevailing cultural values. Health and hospital care can be seen as an institutional activity with its own cultural values, ideas, and knowledge. How the meeting is understood and developed depends on the parties’ previous social and cultural experiences. For the relative to be able to understand and reason with the physician, he or she must be encouraged to actively participate in the social interaction with the physician. This increases the likelihood of developing and improving treatment decisions for the relative together with the patient and of achieving shared goals with the physician (Säljö, 2000; Vygotsky, 1987).

The healthcare organization is a hierarchical system with socially established activities (Rogoff, 1990; Wertsch, 1998). The healthcare organization is characterized by power and dependency relationships between relatives and physicians; relationships that are asymmetrical (Johanson, Sätterlund Larsson, Säljö, & Svärdsudd, 1998; Löfmark & Hammarström, 2005). This asymmetry may affect the result of the meeting because of the rules governing acceptable communication, meaning the level of participation of relatives in the meeting with the physician is determined by the conditions provided by the healthcare organization and the physician (Säljö, 2000; Vygotsky, 1987).

Mishler (1984) underlines the importance of listening to the “voice of the lifeworld,” that is, the statements by relatives and patients about their actual life situation, and of the caregiver basing his or her efforts on this perspective instead of on the “voice of medicine,” that is, the strictly medical and technical information. Other researchers claim that the “voice of medicine” is often the focus of caregivers (Barry, Stevenson, Britten, Barber & Bradley, 2001; Larsson, Säljö, & Aronsson, 1987).

Mediating tools are a key concept of the sociocultural perspective. Vygotsky (1987) and Säljö (2000) describe these tools as expressive, intellectual, linguistic, or psychological tools that can take the form of physical or technical artifacts. Language and thinking are mediating tools of the psychological category. The core of mediated action is that person, context and language interrelate and that learning is achieved jointly with other people. During the meeting between the elderly patient, the relative and the physician, a mediating tool in the conversation is the language.

The physician's position within the organization and his/her ability to express and diagnose medical problems gives him or her power over both patients and relatives. The physician's position of power, for instance to determine the conditions of the meeting (e.g., agenda, setting, and time), may entail that the relatives and patients are not given the opportunity to express their insights or interests. The physician's dominant position can negatively impact the meeting with the relative and the patient (Aronsson & Larsson, 1987; Löfmark & Hammarström, 2005).

Swedish government recommendations (SFS 1982:763; SOU 1997:170; Swedish Government Bill 2005/2006) stress that elderly patients and relatives should be treated in such a manner that their rights to dignity, integrity and safety are upheld. Nevertheless, there appear to be shortcomings in the healthcare organization concerning collaboration and dialogue with patients and relatives (Swedish Government Bill, 2005/2006). Some have argued that physicians are poor at interacting and communicating with relatives and focus too much on the patient (Levine, 2004).

How relatives experience the meeting with the physician in the context of hospital discharge of an elderly patient is an important issue to clarify (The National Board of Health and Welfare, 2008). Therefore, there is a need for more studies exploring the relatives’ experiences in a three-party meeting between relative, elderly patient and physician in the context of medicine and geriatric hospital care in order to identify aspects that may help relatives take up their role in a more efficient manner.

## Purpose

The purpose of this study is to explore relatives’ experiences of meeting with the physician in a hospital setting when an elderly patient is discharged from hospital care to home care, in order to identify aspects that may facilitate relatives in taking up their role in a more efficient manner. The following research questions were used: How is the meeting experienced by the relative? What is of importance for the experience of the meeting (e.g., access to information, opportunity for participation in the meeting)?

## Method

### Design

The study has a qualitative explorative and descriptive design (Silverman, 2001). An inductive and deductive qualitative approach makes it possible to study people's beliefs and experiences by letting them speak about them in their own words (Mishler, 1986). The design is based on the sociocultural theory (Vygotsky, 1987). This approach is based on the view that the meeting is a phenomenon occurring in a cultural and institutional context. This means that the relative's experience of the meeting depends on the context and culture. The sociocultural perspective is based on social interactionism, meaning that the relation between thinking, communication and physical activity is situated. In the social interaction within the specific culture, the actors interpret the meeting with the help of their social and cultural experience. The meeting between relative and physician takes place in a specific institutional setting, namely that of health and hospital care, which affects the contents and structure of the dialogue during the meeting (Junefelt, 2001; Vygotsky, 1987; Wertsch, 1998), as well as the relative's opportunity to discuss and participate in the meeting with the physician. From a socio-cultural perspective, experience and understanding are created through interaction with other people in a social context (Säljö, 2000; Vygotsky, 1987). Experience can be summarized as human experiences that are influenced by the given environment (Hesslefors Arktoft, 1996).

### Setting and sampling

In this study, the definition of “relatives” from the Swedish National Board of Health and Welfare (1998) was used, that is, a relative is a family member, friend, or neighbor looking after a person suffering from any kind of disability or illness. For the inclusion/exclusion of patients, the World Health Organization's (2003) definition of “elderly” was used, that is, someone aged 65 years or over.

The 20 relatives included in the study were adults, living in a large city in Sweden, providing care to elderly patients who had recently been discharged from hospital care to home care. To be included in the study, the relatives had to fit the following three criteria: Swedish speaking; capable of responding to questions individually; have an elderly relative (hereafter referred to as the “elderly patient”) who had recently been discharged from medical—geriatric hospital care and transferred to home care.

Snowball sampling was used as the methodology to get in touch with relatives of elderly patients who had recently been discharged from hospital care to home care (Bryman, 2011).

The author contacted the manager of the primary and home care service to obtain names and telephone numbers of first-line nurse managers in home care, who provided names and numbers for home nurses. These nurses identified elderly patients who had recently been discharged from hospital care and transferred to home care. During a home visit by the home nurses, the patients were asked for verbal informed consent regarding the study's purpose. If the patient agreed, the home nurses sent informal letters explaining the interview study to the patients. Subsequently, the home nurses contacted the patients by telephone to provide additional information about the study and to obtain the patient's approval that the author may make contact.

Subsequently, the author called each patient to ask if any relative(s) had helped during the meeting with the physician prior to their hospital discharge. If a relative had been involved, the author asked for the patient's permission to contact the relative for the purpose of inviting them to participate in the study. In total, 30 patients were contacted and 20 of these had been assisted by a relative. All 20 relatives agreed to be interviewed.

Permission to contact elderly patients who had recently been discharged from hospital to home care, for the purpose of conducting interviews with relatives, was obtained from the manager of the primary and home care service or from first-line nurse managers in home care. The participants were provided with written and verbal informed consent about the study's purpose, its voluntary nature, and the fact that they could at any time cancel their participation. The participants also signed a consent form prior to each interview and they were assured that all data would remain confidential and anonymous (Vetenskapsrådet, 2003).

This study was approved by the appropriate research ethics committee of the Medical Faculty at Gothenburg University.

### Participants

The 20 relatives participating in the study were close relatives providing care to the elderly patient who had recently been discharged from somatic hospital care specialized in geriatrics and medicine, and transferred to home care. The 20 relatives participating in the study comprised nine husbands (aged 65–70), three wives (aged 60–87), four daughters (aged 42–60), two sons (aged 36–40), and two sisters (aged 52–55) in relation to the elderly patient. Three of the 20 relatives had undergone education in healthcare (two nurses and one auxiliary staff).

Of the 20 elderly patients, 11 were women and 9 were men. Their ages ranged from 68 to 95. The average age was 80 (82 for women, 79 for men). The patients had one or more of the following conditions: urinary tract infection, pneumonia, dizziness, respiratory problems or chest pain, and cancer.

### Data collection

The audiotaped semi-structured interviews with the relatives lasted for 30–90 min and were carried out within 2 weeks of the elderly patient being discharged from hospital. The interview guide covered four themes: 1) relative's experiences of the meeting with the physician; 2) relative's expectations of the meeting with the physician; 3) relative's access to information; and 4) relative's opportunity for participation. The themes were not covered in a fixed sequence; instead the interviewer listened actively to the relative and sought to detect nuances and asked appropriate follow-up questions (Silverman, 2001) to avoid misperceptions and misunderstandings, for example, “Please explain what you mean by …?”, “Tell me more about …?”, “Have I understood you correctly when I say …?”.

The interpretation and reduction, into themes and subthemes, of the relatives’ statements are exemplified by quotes enabling the reader to assess the credibility of the result.

### Data analysis

For the purpose of identifying not yet known or poorly known phenomena, the qualitative interview is an appropriate data collection method (Silverman, 2001). The interview study is explorative in order to obtain as much information as possible about the specific problem area (Silverman, 2001).

In such studies, the interviewee and the interviewer meet each other face to face and the interviewer endeavors to understand the interviewee's perspective and experiences (Mishler, 1986). The individual interviews with the relatives is the linguistic tool used to take part of relatives’ experiences of the meeting with the physician in order to identify aspects that may help relatives take up their role in a more efficient manner.

The study's theoretical perspective allows the starting point of the analysis and is dependent on the context. The meeting, that is, the mediating activity, is shaped by the historical, cultural and institutional context (Säljö, 2000; Vygotsky, 1987). The socio-cultural perspective has been used to explain in what way the meeting between the relative and physician in hospital care is obtained.

The primary (inductive) analysis of the material was done using qualitative content analysis (Silverman, 2001, 2006). Qualitative content analysis was considered suitable for making an inductive thematization of the data to obtain a description of the relative's experience of meeting the physician. The secondary (deductive) analysis, that is, themes and subthemes, was analysed using socio-cultural theory (Säljö, 2000; Vygotsky, 1987).

The analysis of the taped interviews was carried out in stages using manifest and latent content analysis to find out what the text said (Silverman, 2001, 2006). In the first stage, all interviews were transcribed verbatim. The interviews in their entirety were used as the initial unit of analysis. All interviews were read several times in their entirety to obtain an overall view of the data (Patton, 2002; Polit & Beck, 2006; Silverman, 2001). With the purpose of the study in mind, the transcribed interview texts were read and key words, expressions, phrases, and sentences, so-called “meaning units” (Silverman, 2001, 2006) were identified and highlighted. The meaning units were labeled with codes related to the content. While identifying the meaning units, all corresponding sections of text were identified, resulting in irrelevant sections in relation to the study's purpose (e.g., complaints about transportation to the hospital or comments about nurses or hospital food) being eliminated from further analysis. This concluded the manifest analysis. In the second stage, the meaning units were compressed to shorten the text while maintaining the original meaning. The remaining sections were used for the continued analysis (and later for selecting example quotes for the results). In the third stage, such key text sections of the interviews linked to the meaning units were grouped according to their content by analysing similarities and differences between the meaning units. Examples of groups of meaning units include: mutual dialogue, satisfactory information, unsatisfactory information, lack of dialogue, empathy and personal meeting. Subsequently, these groups were brought together under tentative themes. For example, the groups of meaning “mutual dialog,” “satisfactory information,” “unsatisfactory information” and “lack of dialog” were brought into the theme “Importance of adapting the communication style.” The themes were then reduced or expanded as a function of what was found during the comparative analysis. This concluded the latent analysis. Throughout this process, questions such as “What stands out?” and “What is seen in the groups?” were continuously asked. The final themes were given suitable headings based on their content (Patton, 2002; Silverman, 2001).

In the secondary analysis communication, power and interaction emerged as the most significant aspects in the meeting between relative and physician. The three aspects were checked against the basic data in order to verify that they did indeed correspond with what had been said in the interviews. These three aspects were subsequently reviewed on the basis of how they can be understood with the aid of sociocultural theory.

## Results

For the purpose of exploring relatives’ experiences of the meeting with the physician in a hospital setting prior to discharge of the elderly patient from hospital care to home care, in order to identify aspects that may facilitate relatives in taking up their role in a more efficient manner, three themes with two subthemes each were identified (Table I).

**Table I:** Themes and subthemes of relatives experience of the meeting with the physician.

Theme	Subtheme
Importance of adapting the communication style	Relatives with healthcare experience take an active role in the meeting and are good at understanding the information recived from the physician Relatives with little or no helathcare experience take a passive role in the meeting and are poor at understanding the information received from the physician
Importance of being aware of the power position	Relatives feel that a positive and respectful demeanor by the physician has a postive impact on their role in the meeting Relatives feel their subordinated role promotes disrespectful demeanor of the physician and negatively impacts the meeting
Importance of time and routines to promote interaction	Relatives become confused about their role and interaction with the physician is negatively impacted when there is insufficient time for the meeting Relatives become insecure in their role and interaction with the physician is negatively impacted when institutional routines are perceived as poor

### Importance of adapting the communication style

The physician needs to plan and create conditions for the conversation in accordance with the needs and resources of the relative. This means the healthcare organization needs to consider this activity as being an important part of the physician's duties. How relatives understood the information received from the physician depended on their familiarity (e.g., education, hospitalization, hospital visits, work) with the health and hospital care environment, which helps relatives take up their role in a more efficient manner. Relatives with little or no experience of the health and hospital care environment tended to rely more on the physician's choices regarding examinations and treatment.

#### Relatives with healthcare experience take an active role in the meeting and are good at understanding the information received from the physician

The relative's degree of understanding of the health and hospital care discourse, and of what was important to discuss, was improved when he or she had health and hospital care experience (e.g., education, hospitalization, hospital visits, work). Relatives with such experience were better prepared, more confident about asking questions, and more able to dialogue with the physician. Physicians seemed to pay more attention to relatives with such experience. Such relatives found it easier to identify any shortcomings on the physician's part with regard to, for instance, provision of information, willingness to listen, and empathy for the relative and/or elderly patient. They also felt more confident about what issues to discuss: “I had some idea of how it worked since I work as an auxiliary. So I was prepared as I knew what I could ask for, I made suggestions.”

Relatives who were familiar with the hospital routines felt prioritized by the physician: “I think one gets more attention when one has healthcare experience.” When the physician spoke in medical terms it was easier for such relatives to act on behalf of the elderly patient and to question decisions: “I didn't trust them [the physicians] so I questioned everything they said … I told them I was a nurse to show that I was qualified to ask the questions I did. I know what people I should put pressure on to get things done. I knew what I could demand.” This greater ease regarding information comprehension and dialog may be explained by these relatives’ experience of the institution, enabling them to understand and interpret the institution's “mediating tools,” that is, its habits and expressions. This means these relatives were prepared as to what to expect from the healthcare institution, which in turn makes the experience easier for both the relative and the elderly patient.

#### Relatives with little or no healthcare experience take a passive role in the meeting and are poor at understanding the information received from the physician

In the opposite situation, meaning the relative had little or no experience of the hospital environment and its routines, the relative was forced to trust the physician's assessment without question. The relative was not part of the institutional activity and could not see the possibilities present in the situation. When the physician failed to adjust the style and vocabulary of the institutional meeting to the relative's abilities and knowledge, it became difficult for the relative to understand the information: “Physicians should be clear when speaking to us relatives; try to speak the same language so we can understand. He [the physician] did not talk normally; just spoke about the medical issues.”

The relatives’ criticism of the physicians’ communication can be related to the institutional social practice whose knowledge tradition and conceptual world are not shared outside the institution. The words used by institutional insiders, that is, by physicians and other healthcare personnel, are not shared by outsiders, that is, relatives and elderly patients. The tools necessary for successful thinking by the outsiders are therefore lacking.

Relatives without hospital experience felt it was difficult to question the care of the elderly patient: “When one doesn't know what to do, who to turn to, it becomes difficult.” Relatives felt that the physicians, with their professional experience, should be able to understand both the relative's and the elderly patient's situation, and to explain things clearly: “The physician could have explained how serious the illness was. I still really haven't understood.” The relatives’ criticism seems to indicate that the voices of the professional and the layman do not always meet. This was a source of dissatisfaction for relatives.

### Importance of being aware of the power position

The physician's ability to show attention, empathy, respect and understanding for the relative affected the meeting and led to either positive or negative feelings.

#### Relatives feel that a positive and respectful demeanor by the physician has a positive impact on their role in the meeting

Analyses showed that when the physician was respectful toward the relative, the latter found it easier to share experiences, insights, and knowledge: “The physician was so understanding, he understood that it was difficult for me.” Such demeanor also fostered mutual understanding and openness.

Physicians with a caring attitude, providing information (e.g., explained the results of tests and examinations) and answering questions in an easily understood manner, used the language as a practical tool to make the relative understand the elderly patient's situation. The manner by which the physician presented and shared the information about a specific treatment could alleviate the negative effects of lacking knowledge.

When the physician was attentive to the relative's questions about the elderly patient's future care and treatment, this increased the feeling of being “seen and accepted,” which in turn led to the relationship between relative and physician working well and brought about a positive experience of the meeting: “The physician spoke to me directly, just like we are now, like a friend. It was better than I had expected so I was surprised. We were given the opportunity to contact the physician if we had more questions, which I appreciated as it was difficult to understand all the information at once.”

Relatives felt it was important for them to be “seen” as this increases their ability to explain the situation to the elderly patient relative, thus being a true resource for the patient.

#### Relatives feel their subordinated role promotes disrespectful demeanor of the physician and negatively impacts the meeting

Sometimes the opposite applied to the meeting between relative and physician: the physician was perceived as being disrespectful toward the relative and the conversation was perceived as being poor. The relative lacked the necessary understanding of the institutional culture and the right tools for the dialogue. This made the relative feel insecure and powerless. “The physicians just did whatever they wanted against my will. That's not right, they should be more considerate. He just wanted me to accept his decision. I wasn't allowed to decide with them.” Such feelings led to doubts regarding tests and treatments.

Relatives also felt that “there was a lack of empathy, an inability to understand how it feels to be a caregiving relative.” The physician was seen to be lacking in respect: “They didn't say much more than NN had to go home and now I can't take care of him anymore.”

If the physician neither explained nor informed, the relatives experienced this as not being accepted and they felt ignored by the physician: “They didn't say much, didn't provide any real information. I didn't really know why she had been hospitalized. It was quite vague. I felt excluded.”

### Importance of time and routines to promote interaction

Relatives felt overlooked by the physician and the healthcare organization because of the lack of time spent on the meeting and the absence of clear routines involving them in the context of hospital discharge. This left them confused, insecure, and worried about the elderly patient's future care.

#### Relatives become confused about their role and interaction with the physician is negatively impacted when there is insufficient time for the meeting

Relatives often felt the physicians were in too much of a hurry and that this showed a lack of planning on behalf of the physician and health and hospital organization, as well as a lack of respect and interest for the relative's insights, needs, and opinions: “Physicians are in such a hurry. They have no time, no information. They are way too stressed. They didn't listen to me, or weren't interested, one feels abandoned.”

Relatives felt that the lack of time reduced the physician's ability to carry out his or her duties in a satisfactory manner: “It all went so fast. Those who decide are in such a hurry. I couldn't keep up. They aren't interested or don't have time.” As it takes time to explain things to a relative, a sufficient amount of time must be allocated to the meeting. If the time is too short, the physician might neglect the relative, who then perceives that “they forgot me.” When physicians stated they had many patients and could not take time to provide more information, relatives felt this was a way for the physician to avoid his or her responsibility.

#### Relatives become insecure in their role and interaction with the physician is negatively impacted when institutional routines are perceived as poor

Relatives had the impression there were no routines for handling hospital discharge. The relative as a “resource” was not always used, and the relative sometimes felt he or she was “just sitting around and waiting, and disappearing within the hospital care system.” Relatives felt it was important to understand the care routines in order to actively assist the elderly patient during the hospital stay and discharge.

Relatives felt overlooked and confused, and became worried about what was about to happen: “There were still questions about what was going to happen. Was she supposed to return [to the hospital] for checkups?”

The relatives felt the care was hampered by the organization's lack of clear routines. The link between hospital and home care did not always work as it should: “Was mother supposed to go somewhere for blood pressure tests, and if so, where was she supposed to go? I was worried and they did nothing to address my concerns and questions.”

The gap between the different care organizations left relatives and patients “in between” and made it difficult for them to convey their message and hampered their ability to convey their insights and wishes.

## Discussion

The purpose of the study was to explore relatives’ experiences of the meeting with the physician in a hospital setting when an elderly patient is discharged from hospital care to home care, in order to identify aspects that may facilitate relatives in taking up their role in a more efficient manner.

In the study, it emerged that relatives with healthcare experience and familiarity with the institutional discourse find it easier to understand the physician's information. Conversely, relatives without healthcare experience found it difficult to understand the physician's information. According to Vygotsky (1987) and Säljö (2000), the language as mediating tool is important for interaction to develop in the specific social activity. For relatives, the environment and culture in the social context may be unknown and frightening, which also impacts the interaction negatively. If the appropriate conditions for successful interaction between relative and physician are not present in the meeting, it becomes more difficult to understand, reason, and act. This absence of interaction can be caused by the physician failing to adapt the institutional conversation's form and vocabulary to the relative's needs. Levine (2004) states that a good physician provides information in a manner favoring comprehension, answers all questions, is available or makes available a knowledgeable substitute, and keeps in mind key pieces of personal history central to the relative's and the elderly patient's unique identities. For some, these things are self-evident; for others, they must be modeled and reinforced within the new realities of healthcare. Being without medical knowledge or insights about hospital routines impacts the relatives negatively when meeting with the physician, as the relative then tends to interpret the information received from the physician incorrectly, based on his or her non-medical and non-healthcare savvy perspective, experiences and expectations (Fiske, 1990; Silverman, 2001). The relative's interpretation of the information conveyed by the physician can either facilitate or hamper the meeting depending on whether or not the relative understands the institutional culture. One insight from the result is that it is important that physicians adapt their communication style. This relates to Selander's (1991) claim that it is important the physician is aware of when, what and how an item of information is to be conveyed, and of the goal of the information transfer. In the healthcare institutional environment, the “voice of the lifeworld” (Mishler, 1984) is not sufficiently heard. On the contrary, the “voice of medicine,” for example, medical terminology, is given room in the meeting. This lack of meeting between the “voices” of the layman and of the professional may explain why relatives feel they cannot fully participate in the dialogue with the physician.

The relatives in this study experienced a feeling of being seen and acknowledged when the physician answered their questions during the meeting. Conversely, they experienced a feeling of being ignored and dissatisfied when the physician did not involve them in decisions related to the elderly patient's discharge from hospital. According to a sociocultural perspective, this can be a result of people perceiving situations differently in light of their earlier experiences (Säljö, 2000). Therefore, there is a risk that relatives can perceive the meeting negatively in situations where the physician has not had a chance to, or made efforts to, develop a relationship with the relative. Another reason that can make relatives perceive the meeting with the physician negatively may be that relatives do not have enough information to understand the physician's perspective.

The physician, being in his or her professional environment, is well familiar with the setting of the meeting, while the relative is in more unfamiliar territory, which naturally creates an imbalance in the relationship. The meeting is dominated by the physician, in that he or she determines the agenda, speaks the most, initiates lines of discussion, contributes the key statements, summarizes and concludes the discussion (Agar, 1985; Silverman, 2001). It is therefore important the physician is aware of his or her power position in the meeting with the relative and the elderly patient.

The relatives’ statements indicated that the interaction between them and the physician was hampered by the physician's apparent lack of planning. In order to improve the quality of the meeting between relative and physician, the relative needs support and assistance from the physician to gradually obtain understanding of the situation and to make the relative feel part of the institutional environment. These findings can be related to Andershed and Ternestedt's (2001) description of relatives’ participation in care as “involvement in the light” or “involvement in the dark.” Involvement in the light is characterized by a trusting relationship between relatives and health professionals. Involvement in the dark is characterized by insufficient interaction and collaboration, meaning the relative is not acknowledged by the staff, but instead must maneuver in the dark when trying to help the patient. Interaction as an activity process (Säljö, 2000) for relatives’ learning is determined by the social setting, culture and historic influences. The physician is an important actor in the activity process for information to become understandable and adapted to the relative's experiences, knowledge and ability. The relative thereby becomes a mediating tool that offers safety and support when interacting with the physician to agree on future care and treatment for the elderly patient. According to Schweitzer et al. (2005), the family plays an important role in making decisions about patients. Physicians should therefore encourage relatives to express their views and to contribute their experiences during the social interaction in order to reduce misunderstanding and frustration.

The result showed that relatives wanted to participate in the meeting with the physician, but that physicians were not always perceived as being willing to involve the relatives. It may be that physicians feel uncomfortable in the presence of relatives, especially if the relative has a “negative” effect on the elderly patient (e.g., marginalizes the patient; Ho, 2008). It may be that the relative's concerns regarding the elderly patient in terms of health and treatment (Li, 2005), as well as regarding the workload of being a caregiver (Jansson, Nordberg, & Grafström, 2001; Levenstein, McCracken, McWhinney, Stewart, & Brown, 1986), makes him or her apprehensive, even “defensive”, about and during the meeting with the physician. The relative's situation could be improved if the physicians were to make an effort to understand the relative's situation, perhaps by offering the relative a chance to discuss the discharge process in terms of the elderly patient's well-being and health. In some cases it may be beneficial for the physician and relative to meet without the elderly patient. In a study by Karnieli-Miller, Werner, Neufeld-Kroszynski and Eidelman (2012) it emerged that “triadic” communication is actually a series of alternating dyadic exchanges during which the third person attempts, more or less successfully, to actively participate. During the introduction and summation the core dyad moves from physician–patient to physician–companion. These shifts may represent emotionally trying role transformations from that of companion to that of caregiver.

The relatives were under the impression that there were no clear organizational routines for how discharges are handled and how to plan for and behave during the meeting with the relative, which had a negative impact on the physician's behavior, thereby contributing to the relative's feeling of confusion and insecurity about what was about to happen. One explanation put forward is that there are obstacles between different health and hospital care organizations. The link between hospital and home care does not work as it should. The gap between the different care organizations leaves relatives and patients “in between” and makes it difficult for them to convey their message. These findings can be related to the study by Rydeman, Törnkvist, Agreus and Dahlberg (2012) who found that older persons and their relatives in these situations could easily be lost in an in-between experience in which their existence is threatened. Organizational constraints had a negative impact on the relationship and trust between relative and physician, and made it difficult for relatives to convey their message and hampered their ability to convey their insights. It is therefore important that the physician is aware of the importance of time and routines as factors promoting interaction with the relative.

Petek Ster (2012) found that early clinical exposure helps medical students develop positive attitudes toward their learning, future medical practice and the importance of improving communication skills in the meeting between patients and physicians. Karnieli-Miller et al. (2012) believed that physicians need (training in) specific communication skills, for example, clarifying the rules and structure of the conversation for effective and empathic handling of triadic communication situations without interruption and frustration.

This study found that the communication style, with its underlying power relationship and quality of the interaction, was crucial for information exchange to be achieved in the meeting. How relatives experience the communication is determined by the physician's power position as it appears through his or her demeanor. The interaction between relative and physician is essential for there to be a respectful meeting and exchange of information. By giving relatives a chance to participate in the meeting and express their experiences and knowledge, physicians can add value by helping the elderly patient understand his or her illness and by apprising the physician of the patient's situation. By so doing, the relative becomes a tool for knowledge exchange between physician and patient; this contributes to creating understanding and meaning. However, healthcare routines and the physician's power position make it difficult for relatives to get involved in the care. In the absence of the right conditions (e.g., the physician offers the relative space to express his or her views and uses a language the relative can understand) in the meeting between the relative and the physician, the dialogue, interaction and involvement all suffer, at the expense of both the relative, the physician, the elderly patient and the healthcare system.

## Strengths and limitations of the study

Relatives can be an important asset and resource for the elderly patient. The role as care-providing relative can entail a heavy workload and be unclear for all those involved in the healthcare situation, that is, the elderly relative patient, the healthcare practitioners, and the relative.

As all of the relatives invited to participate in the study voluntarily agreed to participate, there is no risk of bias due to a situation of only the most interested relatives being willing to participate. It is believed that the experiences of the 20 relatives contributed a rich and comprehensive body of material.

The author's description of the interviewed relatives’ experiences can be limited by the fact that at the time of the interview the relative might not fully express what he or she wanted to say. As a result, the relatives’ statements remain a fragmented picture of their experience. To enhance the communicative credibility during the interview, respondent validation (Silverman, 2001) was used, that is, the respondent validation was tested by means of dialogue with the relative. To avoid misunderstandings during the interviews, summations were discussed to offer the relative the opportunity to make corrections or clarifications.

The researcher's background in nursing can be an advantage when understanding and interpreting the relatives’ descriptions of the meeting with the physician. On the other hand, this familiarity may lead to nuances in the descriptions being lost. To avoid this potential problem, attention was constantly paid to maintaining a conscious and critical approach to understanding.

The author's experience in elderly care nursing is believed to have facilitated understanding of the relatives’ expressions of meanings of participation in the meeting with the physician. To offset the problem that analysing an interview text always involves the analyser's interpretation (Silverman, 2001), and the fact that analysis might be influenced by the researcher's prior understandings of the topics involved, quotations from the interviews have been used to illustrate the results and to strengthen the analysis.

Discourse analysis could have been an alternative method for analysing the interviews made within the framework of this study. As the author did not want to focus on power, the socio-cultural perspective was chosen instead. However, with the results at hand, one notes that power was indeed a dominant theme and that future studies in this area may find it worthwhile to focus on how power is mediated.

The participants in this study were all Swedish speakers. One may wonder how immigrants, with their different languages and who are a growing group in Sweden, experience the situation considered in this study. To facilitate for this group of patients and relatives, an interpreter can be helpful during the meeting with the physician. This is a patient right according to Swedish legislation. However, the healthcare organization does not always live up to this demand (Hedemalm, Schaufelberger, & Ekman, 2007).

## Conclusions

The purpose of this study was to explore experiences of relatives’ of the meeting with the physician in a hospital setting when an elderly patient is discharged from hospital care to home care, in order to identify aspects that may facilitate relatives in taking up their role in a more efficient manner. Despite Swedish rules and legislation referring to the need to involve and communicate with relatives during an elderly patient's treatment and care, this study showed that participation and communication are lacking when the relative meets the physician in connection with the elderly patient's discharge from hospital. The relatives’ experiences of limited participation and insecurity in their role seem to depend not only on the physicians’ shortcomings but also on organizational obstacles concerning collaboration, dialogue and information. In order to increase the participation of relatives and to help them take up their supporting role, development in terms of both the healthcare organization and the skills of physicians is needed. To improve the quality of the meeting between relatives and physician, the relatives need more support and assistance. The physician's professional communication skills, including information, respect and collaboration, need to receive more attention from both their medical educators and their healthcare organization. This study could be used to demonstrate the relatives’ feelings as regards the importance of being an active participant in the meeting.

To increase the relatives’ participation in the communication during the meeting with the physician, it is necessary to instill a feeling of safety and trust. One can attain this by starting the discussion with the individual's particular situation and needs. A trustful relationship can be established by making relatives feel welcome and motivated to participate in the discussions. It would therefore be of interest to develop courses for physicians, within the framework of their medical school curriculum, that include advice on how to communicate, both dyadic and triadic meetings, with and give more guidance and support to relatives who attend meetings to decide on an elderly patient's care.
